# Supplementary material for: Genetic diversity in terrestrial subsurface ecosystems impacted by geological degassing
Source: Nat Commun. 2022 Jan 12;13:284. doi: 10.1038/s41467-021-27783-7 (PMC8755723; doi:10.1038/s41467-021-27783-7)
Supplement: Supplementary file 1 — Supplementary Information [file 41467_2021_27783_MOESM1_ESM.pdf]

Main Supplementary File for:

Genetic diversity in terrestrial subsurface ecosystems impacted by geological degassing

Till L.V. Bornemann[1], Panagiotis S. Adam[1], Victoria Turzynski[1], Ulrich Schreiber[2], Perla Abigail Figueroa-Gonzalez[1], Janina Rahlff[1#], Daniel Köster[3], Torsten C. Schmidt[3], Ralf Schunk[4], Bernhard Krauthausen[5], and Alexander J. Probst[1,6*]

1: Environmental Microbiology and Biotechnology, Faculty of Chemistry, University Duisburg-Essen, Germany
2: Department of Geology, University Duisburg-Essen, Germany
3: Instrumental Analytical Chemistry and Centre for Water and Environmental Research (ZWU), University of Duisburg-Essen, Germany
4: Geyser-Center, Andernach, Germany
5: Institute of Applied Geosciences, Karlsruhe Institute of Technology, Germany
6: Centre of Water and Environmental Research (ZWU), University of Duisburg-Essen, Universitätsstraße 5, 45141, Essen, Germany

[#]Present address: Centre for Ecology and Evolution in Microbial Model Systems (EEMiS), Department of Biology and Environmental Science, Linneaus University, Kalmar, Sweden

*To whom the correspondence should be addressed:
Alexander J. Probst
Environmental Microbiology and Biotechnology, Faculty of Chemistry, University of Duisburg-Essen
alexander.probst@uni-due.de

Content:

1. Supplementary Methods
2. Supplementary Discussions
3. Supplementary Figures
4. Supplementary Tables
5. References

1. SUPPLEMENTARY METHODS

Estimations of annual total erupted carbon and intracellular erupted carbon. The annual total erupted carbon was calculated based on the available CO_2 and HCO_3 concentrations and the eruption volume (Table S1) and the average estimate of the intracellular carbon amount from Kallmeyer et al. (2012)[1] of 14 fg $cell^{-1}$. During tourist season (April 1 – October 31, ~ 210 days, rest of the year no eruptions), three eruptions are initiated per day with an average eruption volume of 6.5 m^3. We used equation Eq (1) to calculate the total erupted organic carbon and Eq (2) to calculate the total erupted cellular carbon.

Eq (1) $T_{otal\ carbon}=V \times E \times D \times (CO_2 \times F_{CO2}+HCO_3 \times F_{HCO3})$=6,270 kg carbon/year

Eq (2) $Cell_{ular\ carbon}=V \times E \times D \times C \times$ cellular carbon = 111.475 g

Abbreviations	Description	Value
V	eruption volume	6.5 m^3
E	number of eruptions per day	3
D	tourist season length in days	210 (7 months)
CO_2	concentration of CO_2	1500 mg / l
F_{CO2}	mass fraction of C in CO_2	0.27
HCO_3	concentration of HCO_3^-	5700 mg / l
F_{HCO3}	mass fraction of C in HCO_3^-	0.20
C	cell concentration	3.5 x 10^6 cells ml^{-1}

Phylogenetic placement of draft genomes. Marker genes *rpL16*, *rpL18*, *rpL2*, *rpL22*, *rpL24*, *rpL3*, *rpL4*, *rpL5*, *rpL6*, *rpS10*, *rpS17*, *rpS19*, *rpS3* and *rpS8* were identified in the draft genomes using blastp with an e-value cutoff of 0.01 against a previously described database of bacterial homologues to these marker genes[2] and verified by blasting of the resulting candidate sequences against all proteins of all genomes in the database with an e-value cutoff of 10^{-6}.

Metagenomic binning. Scaffolds of each sample were binned using ABAWACA[3] and ESOMs[4] based on tetra-nucleotide frequency of DNA fragments of 3 kbp and 5 kbp as minimum length and

5 kbp and 10 kbp as respective maximum length cutoffs, respectively. *Escherichia coli* (low GC%) and *Streptomyces griseus* (high GC%) spike-in genomes prior to nucleotide frequency calculation were used to verify the success of the ESOM training and only ESOMs showing a good clustering of these controls were used for binning. SulCav samples were not binned using ESOM due to sample size. Differential coverage binning was achieved using Maxbin2[5] (default parameters), if multiple samples were available for the same ecosystem. GA samples were additionally binned using CONCOCT[6] (default parameters). The different binning results were aggregated using DAS Tool[7] (default parameters) and the genomic bins were curated based on their GC content, coverage and taxonomy. Completeness and contamination was assessed using a previously described set of universal 51 bacterial and 38 archaeal single copy genes[8] and only genomes with >= 70 % completeness and <= 10 % contamination were used for further analyses. Genomes originating from different samples of the same ecosystem were dereplicated using dRep[9].

Metabolic potential predictions. Hidden Markov-models (HMMs) and their corresponding score thresholds originating from Anantharaman et al. (2016)[10] were used to identify key enzymes in chemolithoautotrophic pathways (see Supplementary Table 8 for a list of HMMs, their respective score thresholds and the pathways they are indicative of). If multiple genes indicative of a single pathway were recovered in assemblies, their abundances were aggregated by determining the maximum abundance. For the prediction of the metabolic potential for sulfur oxidation / reduction on genomes, many of the included pathways require specific genes to be absent while others need to be present to indicate the presence of the specific pathways. Thus, we used the following presence/absence scheme for the HMMs detailed in Supplementary Table 8 to identify present pathways in genomes.

Sulfide oxidation	*sulfide_quinone_oxidoreductase_sqr* OR *fccB* need to be present
Sulfite reduction	*dsrA* AND *dsrB* AND *dsrD* need to be present
Sulfur oxidation with *dsr*	*dsrA* AND *dsrB* need to be present but *dsrD* needs to be absent
Sulfur oxidation with *sor*	*sor* needs to be present
Sulfur oxidation with *sdo*	*sdo* needs to be present
Sulfate reduction with *asr*	*asrA* AND *asrB* AND *asrC* need to be present
Sulfate reduction with *aprA* and *sat*	*aprA* AND *sat* need to be present
Thiosulfate disproportionation	*phsA* needs to be present

Geochemical measurements of metagenomic samples.

Temperature. The air temperature was measured using a mobile phone and the water temperature using thermometers in triplicate.

pH. The pH was measured using pH strips with 0.2 Interval coloring schemes in triplicate.

Sulfide. 200 ul of water were added to 1 ml of 1 % (w/v) Zinc-Acetate and mixed on-site. Concentrations were measured according to a modified version of the protocol by Cline[11] in triplicate. 20 µl of sample was added to 400 µl of 1 % (w/v) Zinc-Acetate and 0.2 % (v/v) acetic acid. 25 µl of both 0.5 % (w/v) ferric ammonium sulphate + 0.096 % (v/v) sulfuric acid and of 0.2 % (w/v) 4-amino-N,N-dimethylaniline sulphate + 19.6 % (v/v) sulfuric acid were added to each well of the plate reader, followed by addition of 100 µl of diluted sample. The plate was incubated in the dark for 30 minutes followed by detection of absorbance at 664 nm. The sulfide concentration was determined using a dilution series of 0.05 mM to 2 mM of sulfide. Measurements were performed in triplicate and triplicates were averaged.

Fe(II) determination. 100 ul of sample were added to 900 ul of 1 M HCl and mixed on site. Fe(II) concentration was determined by addition of 20 µl of sample to 180 µl of 0.1 % (w/v) ferrozine with 50 % (w/v) ammonium acetate in plate reader plate wells, incubation in dark for 15 minutes

followed by absorbance detection at 560 nm. A dilution series from 0 mM to 100 mM was used to determine the concentration.

Total iron determination. 100 ul of sample were added to 900 ul of 1 M HCl and mixed on site. 100 µl of the mixture (as well as the dilution series of Fe(II) concentration determination) were added to 900 µl of 10 % (w/v) hydroxylamine-HCl in 1 M HCl and incubated on a shaker for 15 minutes to dissolve iron precipitates. Then 20 µl of solution are added to 180 µl of 0.1 % (w/v) ferrozine with 50 % (w/v) ammonium acetate in the microtiter plate and the absorbance is detected at 560 nm.

Total organic carbon (TOC) measurements. 2 ml of 1 M HCl were added to 10 ml of filtered sample (0.45 um pore size) and degassed by bubbling air into the vials for 15 min to purge the sample of inorganic carbon. The TOC concentrations were determined using a TOC-L (Shimadzu). Two biological replicates were measured with three technical replicates.

Ion measurements. 0.1 um pore-size filtrated water was used for ion measurements. Samples were measured in dilutions of 1:100 and 1:600 in distilled water to be able to accurately quantify both highly abundant ions and lower abundant ions. Two biological samples were analyzed using the Dionex Aquion ion chromatography system (Thermo Scientific, USA). Anions were analyzed with a Dionex IonPac AG23-4 µm guard column, a Dionex IonPac AG23-4 µm 2 x 250 mm analytical column as well as an AERS 500 Carbonate 2 mm suppressor, Ultimate 3000 heating element and a DS6 heated conductivity cell detector. Cations were measured on a CS12A RFIC 2x 250 mm analytical column and a CG12A RFIC detector. Measurements were done in technical triplicates and distilled water blank concentrations were subtracted.

Long-term geochemical measurements were performed according to German TrinwV-GW (drinking water guidelines).

DAPI Staining and cell enumeration.

Staining. Water was filtered on-site through 0.1 um pore size filters. Filters were incubated with 10 ug/ml DAPI in 2 % (v/v) Formaldehyde for 5 minutes, washed with 30 ml of distilled water, dried for 10 minutes and stored in the dark at 277 K till use. All steps involving DAPI and stained filters were done in the dark.

Enumeration of cells. Cells were quantified in cells ml^{-1} using the Axio A.1 epiflouorescence Microscope by enumeration of the cells in ten horizontal and ten vertical fields, extrapolating counts to the entire filter area and normalizing through the filtered volume.

2. SUPPLEMENTARY DISCUSSION

Extended discussion on geography and dispersal of Altiarchaeota

All Alti-1 species (including two new bins from GA and SulCav) placed the under the *Ca.* Altiarchaota subclade Alti-1 based on our phylogenomic analysis. Alti-1 organisms are known to dominate their respective subsurface ecosystems and form biofilms using their characteristic hook-like hami surface appendages[12,13]. Our phylogenomic analysis reproduced prior findings by Bird[14] and expanded upon the Alti-1 subclade phylogenetic diversity. Based on our analysis, Alti-1 genomes have an extreme degree of provincialism, with clear clustering by continent of origin (North America, Europe, Asia), which is also reproducible based on ANI and AAI as estimates of genome similarity. Prior intercontinental studies have also observed a continental clustering for their respective *Synechococcus*[15], *Sulfolobus*[16] or *Comamonas testosteroni*[17] assemblages and identified geographical distance as the main predictor as opposed to other environmental parameters[15,16].

The relationship between genomic similarity and geographic distance is in general a well-known occurrence in nature and as a consequence, concepts like the isolation-by-distance theory[18,19,20] and others[21,22] have been developed. Generally, isolation-by-distance suggests that distance to be the main barrier for dispersal and consequently limiting the exchange of genetic material. Such relationships are also well-established for microorganisms, with atmospheric and hydrological phenomena contributing to the dispersal[23,24]. However, microorganisms hitchhiking on plate tectonics for their dispersal is not unheard of either[25,26]. Since *Ca.* Altiarchaeales are anaerobic subsurface dwellers, surficial dispersal mechanisms or subsurface dispersal via oxygenated groundwater systems are unlikely to have contributed to their spread across multiple continents. Their hami, have been shown to be very adhesive to a variety of different surfaces, in

addition to being rather heat- and pH-stable, thus likely anchoring *Ca.* Altiarcheales biofilms in their location[12] and might restrict their dispersal mechanism to plate tectonics.

Geological reasoning behind the enhancement of microbial activity in ecosystems affected by geological degassing

In our study, we observed that microbes in the Geyser Andernach and in the Crystal Geyser showed similar replication measures to near-surface ecosystems, based on the observed correlation between bacterial replication indices as estimated using iRep and the sampling depth and we proposed that the higher nutrient flow rates present in these ecosystems could very well explain this difference. In the following, the geological reasoning behind the proposal will be explained in more detail.

In sediments, aquifers flow through pore channels in the subsurface and their amount generally decreases with depth as the sediment becomes more compact and the pores mineralize, thus reducing the diameter and consequently the flow rate. Joint aquifers, which are characterized by solid rock formation-embedded channels, can allow for much greater flowrates that can be up to multiple magnitudes greater than sediment pore channel aquifers since no compression occurs. In aquifers, the nutrient availability, provided they are dissolved in groundwater, is directly proportional to the flow volume and thus the greater flow rate in joint aquifers should automatically increase the microbial activity since nutrient availability is the generally the growth limiting factor in the subsurface[27].

Ecosystems like the Geyser Andernach or mofettes that are affected by mantle degassing are such joint aquifer systems since the release of the CO_2-rich gases requires complex channel systems with a mixture of water and CO_2 with traces of, e.g., hydrogen, SH_2, methane and nitrogen.

In the lower crust, organic products are generated by the combination of high pressure and temperature comparable to the Fischer/Tropsch-Synthesis[28]. These products are mainly long-chain alkanes depending on the depth, gas composition and availability of metals on mineral surfaces. Below 20 km depth, both water and CO_2 are supercritical and infinitely mixable. Decreasing pressure liquifies the water, segregating the supercritical CO_2 and causing it to rise to the surface as droplets, capturing the nonpolar alkanes in its current. Once CO_2 becomes subcritical, the organics precipitate at the interface since the gaseous CO_2 cannot keep them in solution. Gaseous CO_2 then travels on to the surface and initiates a current, transporting the organics. This current results in a steady supply of organics as well as gases like CO_2, H_2 and N_2, fueling microbial activity and replication and is thus a probable explanation for the comparatively high replication measures in high CO_2 environments.

Possible factors influencing iRep as a measure of bacterial replication

Peak-to-Trough ratio estimators like iRep measure the difference of the abundance of the genome close to the origin of replication and the terminus of replication. From this ratio, they infer how many replication forks are in progress across the investigated population. Thus, iRep values give a quantitative estimate for the number of replication forks in progress for a given population (represented by a given genome) in a given sample (represented by the mapping of reads onto the given genome). While a high value of this measure does give an indication that a population is actively replicating as opposed to dormant, it is no measure of activity in a strict sense as the measure does not have a per time unit component and thus no estimation about the speed of the measured ongoing replication can be made.

To ensure that iRep values are valid, some strict requirements are made for both the mappings and the genomes analyzed with the iRep algorithm. These include a minimum average genome coverage greater than five, less than 175 fragments per Mbp (and only scaffolds >= 5kbp are used by iRep), a minimum of 98 % of windows kept (iRep calculates a mean coverage for 5 kbp windows and if those are empty, they are discarded), an R^2 >= 0.90 for the slope calculation of Peak-to-Trough ratio and finally that there is no correlation between GC content and coverage after GC correction. All values reported herein met these requirements. Despite this, quite a few factors can influence the mapping of reads to the genomes and those in turn can influence the iRep values. One such factor are very closely related species where the reads cannot be unambiguously assigned to a specific genome and thus by default get randomly assigned if multiple alignment locations with identical scorings are found. We mitigated this effect by using dRep[9] to dereplicate genomes if multiple samples from the same ecosystem were analyzed. Genomes originating from the same sample should on the other hand be sufficiently distinct as they would otherwise not assemble as separate scaffolds. As a second factor, ubiquitously occurring horizontal gene transfer (HGT) or mobile genetic elements can also confound iRep measures as they can result in the ambiguous assignment of reads. Requirements of the iRep algorithm attempt to identify problematic mappings by, e.g., having the R^2 >= 0.90 requirement for the Peak-to-Trough ratio calibration curve. Minor perturbations like HGT might be able to pass the iRep requirements. Consequently, this study did not use iRep as difference measure between populations of individual species, it rather gathered iRep values across 895 genomes and categorized them based on sampling depth to perform univariate statistics. The large number of genomes and their categorization should account for minor inconsistencies arising from HGT and other confounding genomic features.

3. SUPPLEMENATARY FIGURES

Supplementary Figure 1 | Diagram of the subsurface environment of the Geyser Andernach. The upper 83 m of the geyser well have a casing and cement sealing, ensuring that only subsurface water enters it. The residual length of the borehole (83-351.5 m) is intermittently covered by bridge-slotted screens through which CO_2-saturated water (and microbes) can enter the geyser system. The bridge-slotted screen segments are positioned at 85-105 m, 110-120 m, 145-150 m, 185-195 m, 200-205 m, 210-225 m, 230-235 m, 240-260 m, 265-275 m, 290-295 m, 305-310 m, 315-325 m and 330-335 m, respectively. Microbes are expected to reside on every surface of the ecosystem, including faults and quartz vein system as well as in the sediment. SW indicates South-West and NE indicates North-East.

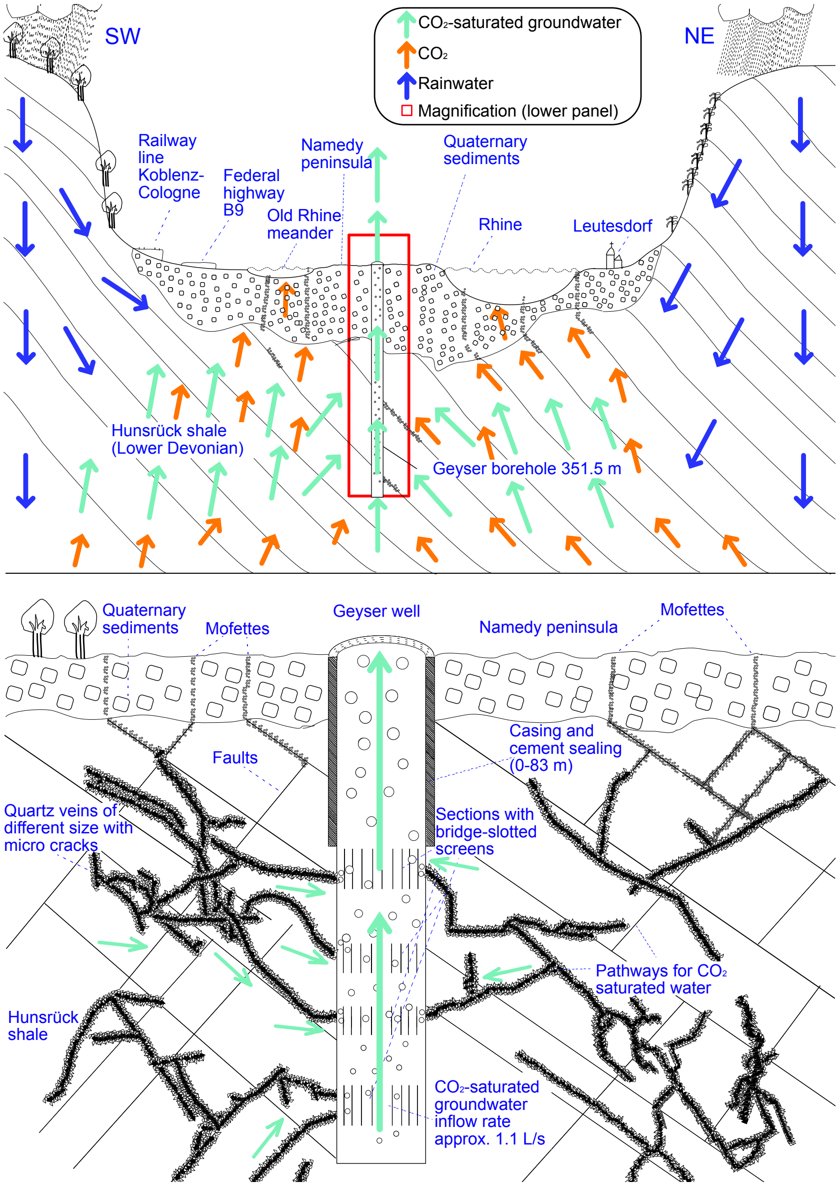

Supplementary Figure 2 | Estimation of metagenomic coverage and sequence diversity. Colors indicate different samples. The curve was estimated using Nonpareil3[29] with kmers of size 20. The circle indicates the estimated metagenomic coverage at the sequenced sequencing depth, i.e., how much of the diversity is covered at the used sequencing effort. This estimated coverage is slightly above 80%, indicating that more than 80% of the diversity is covered by the metagenomes. For the samples GA_E11, GA_E12 and GA_E21, respective estimated diversities are 16.711, 16.534 and 16.732, indicating that the community complexities did not change between the eruptions. Please note that the blue curve is overshadowed by the red curve as they are highly similar.

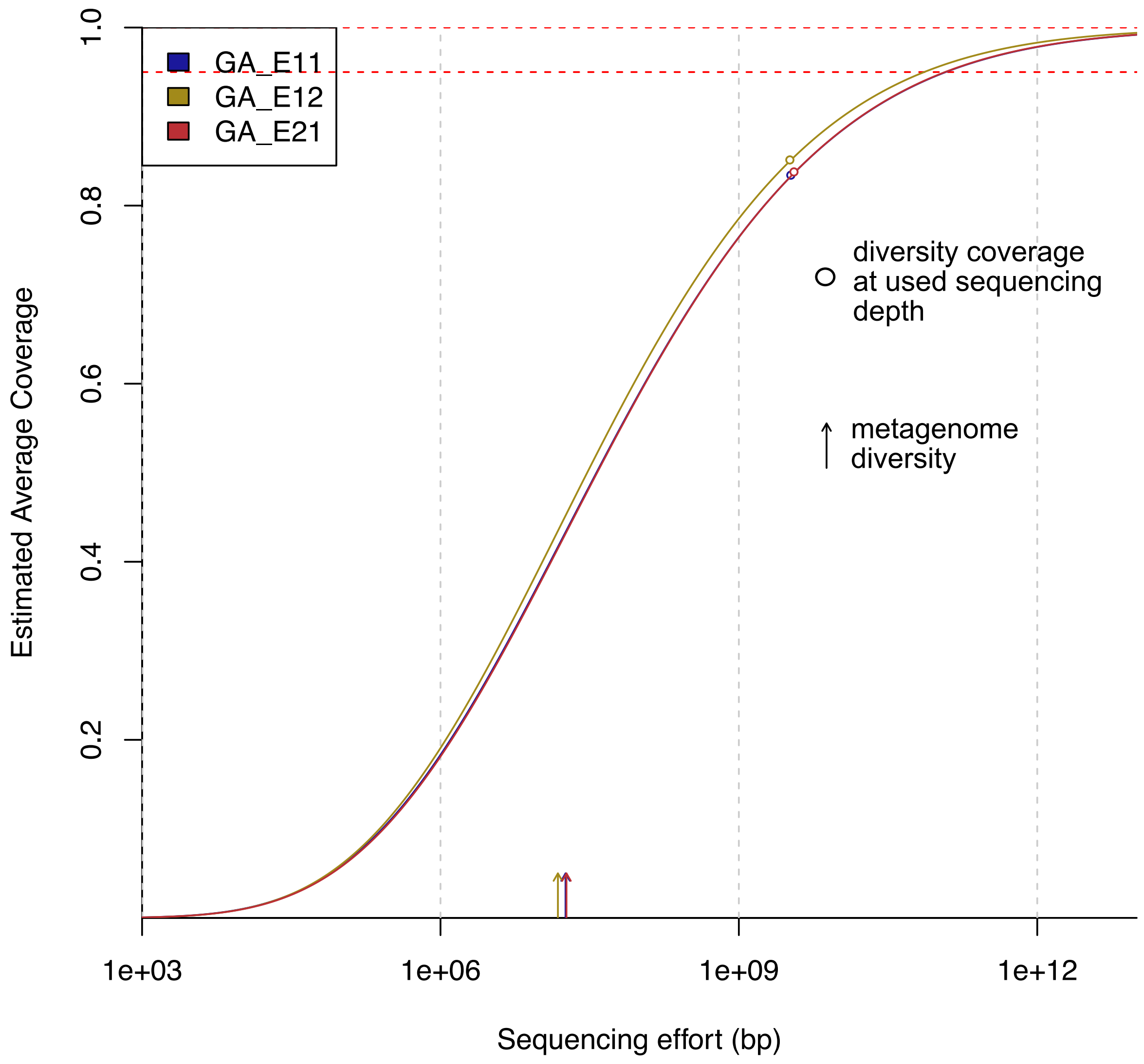

Supplementary Figure 3 | Cell densities in Geyser Andernach in comparison to other sample types and depths. Cell concentrations across different sample types and depths from Magnabosco et al. (2018)[30] (colored dots) are set in relation to the concentration of the Geyser Andernach (black dot, mean cell concentration is shown). Colors are used to additionally highlight the separate sample types. To increase visibility of the Geyser Andernach sample, only cell concentrations of ecosystems with 0-500 m depth and with relevant sample types are shown.

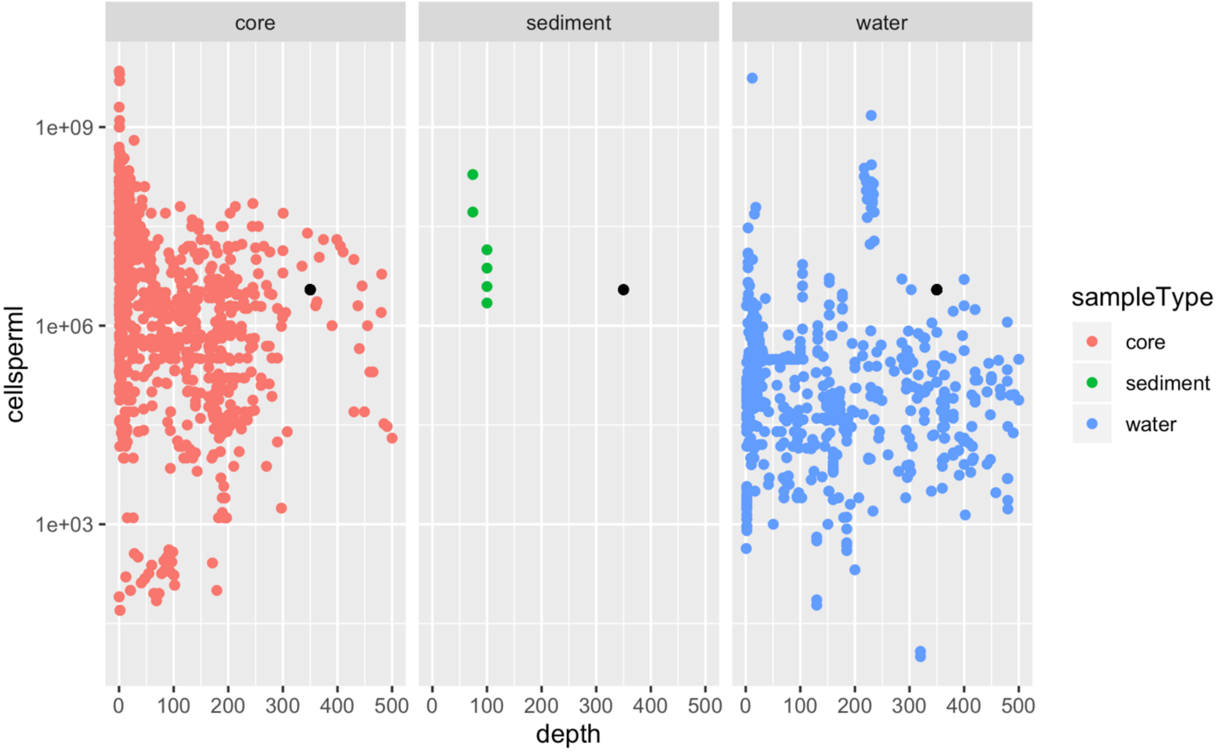

Supplementary Figure 4 | Comparison of maximal growth rates and in situ replication indices to sampling depth. Maximal growth rates were accessed using growthpred[31]. Growthpred values are plotted in the top two panels, either with all doubling times or with only doubling times <=5 h as codon usage bias utilizing methods underestimate the growth rate for very slow growing organisms (defined as doubling times > 5 h)[32]. For genomes from the Crystal Geyser (CG), the sample depth of the eruption phase was used where the genome was enriched[8]. If a genome was not enriched in any specific phase, the average depth of the three CG eruption phases (267 m) was used instead. Pearson correlation values and their p-value are depicted on top of the respective panels. Black dots indicate growthpred/iRep values of individual genomes and the red dot indicates the median value per depth. The exact p-values from top to bottom panel are: p< 2.2x10^-16 (minimal p-value in R), p = 0.001048 and p=6.258x10^-08, respectively.

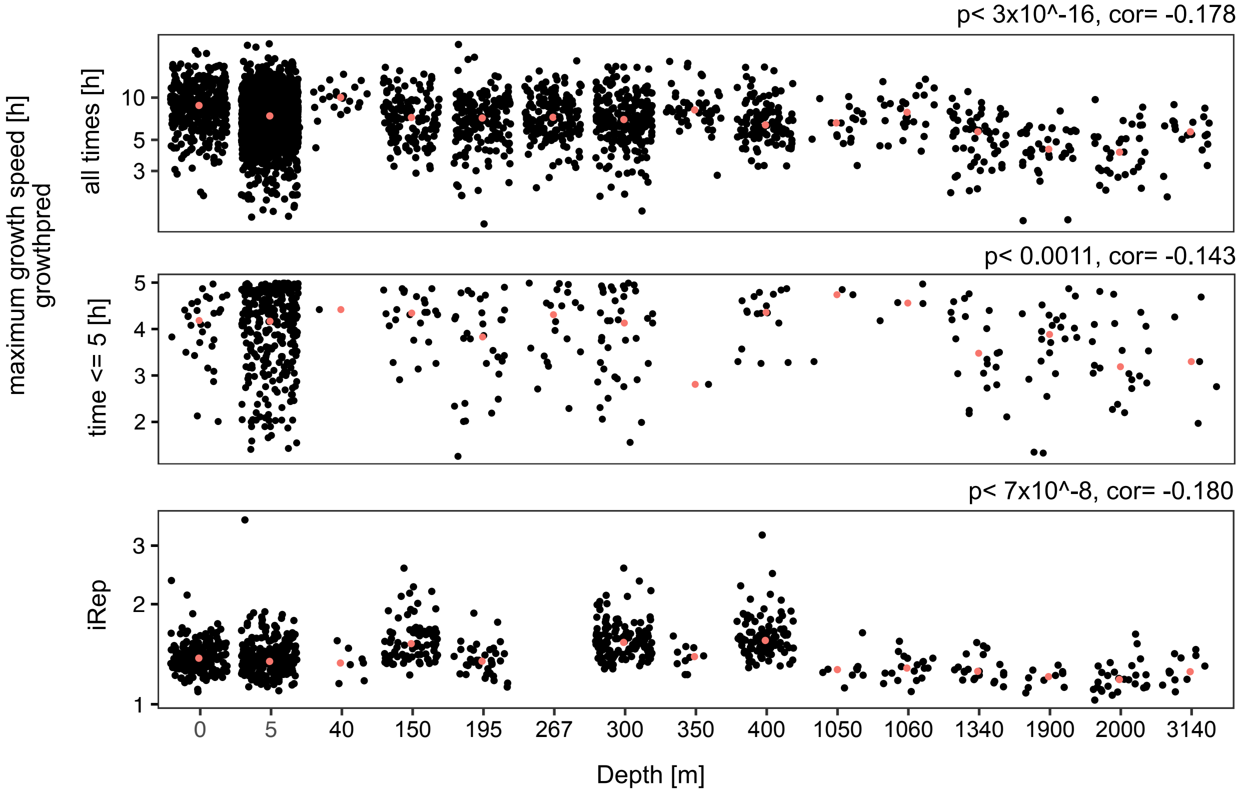

Supplementary Figure 5 | Chemolithoautotrophic metabolic potential across ecosystems. The heatmap shows the number of HMM hits per metagenomic assembly, with the pathways as well as the individual genes being depicted on the Y-axis. Colored squares on the top depict the sample type. If multiple biological replicates of samples were available, up to three were depicted. Sample order is according to Figure 3 and Supplementary Table 5.

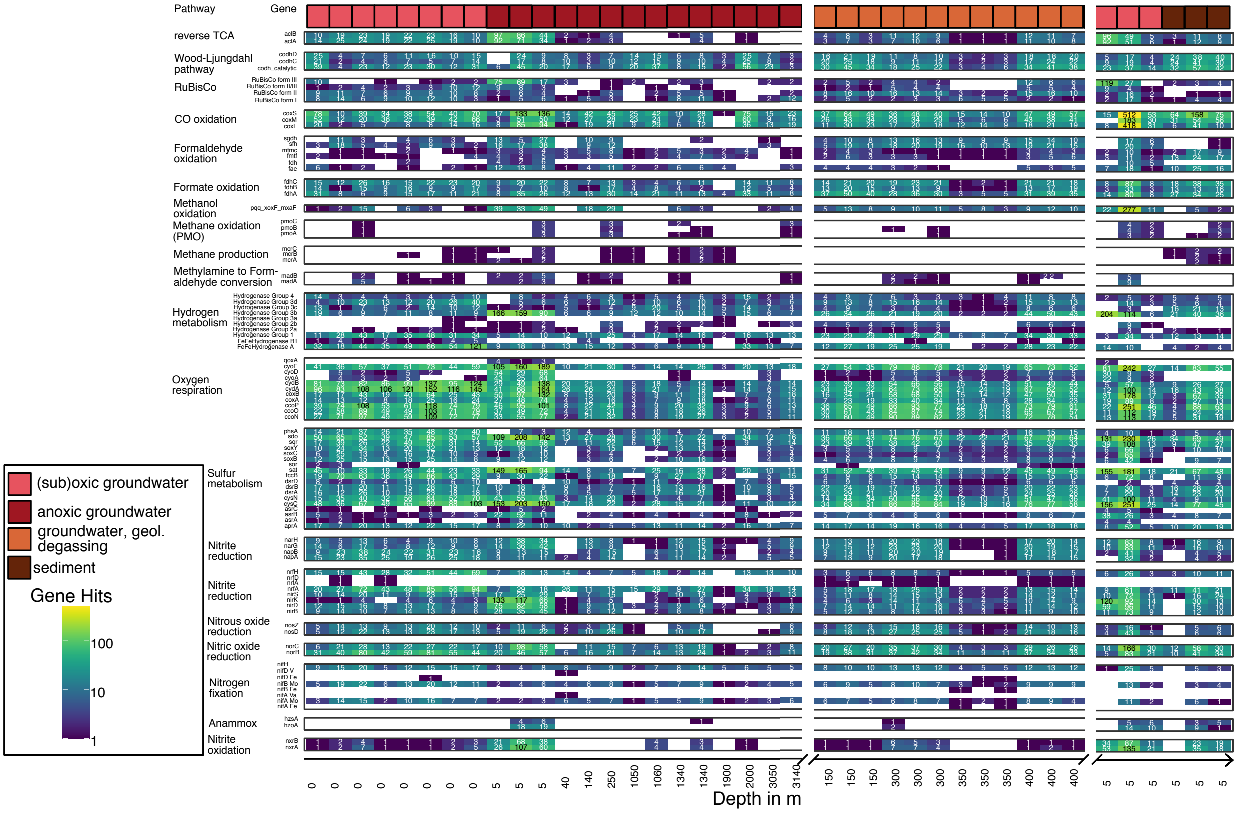

Supplementary Figure 6 | Chemolithoautotrophic metabolic potential across ecosystems|. The heatmap shows Z-scaled normalized abundance of key enzymes involved in chemolithoautotrophic pathways, with the pathways as well as the individual genes being depicted on the Y-axis. Colored squares on the top depict the sample type. If multiple biological replicates of samples were available, up to three were depicted. Sample order is according to Figure 3 and Supplementary Table 5.

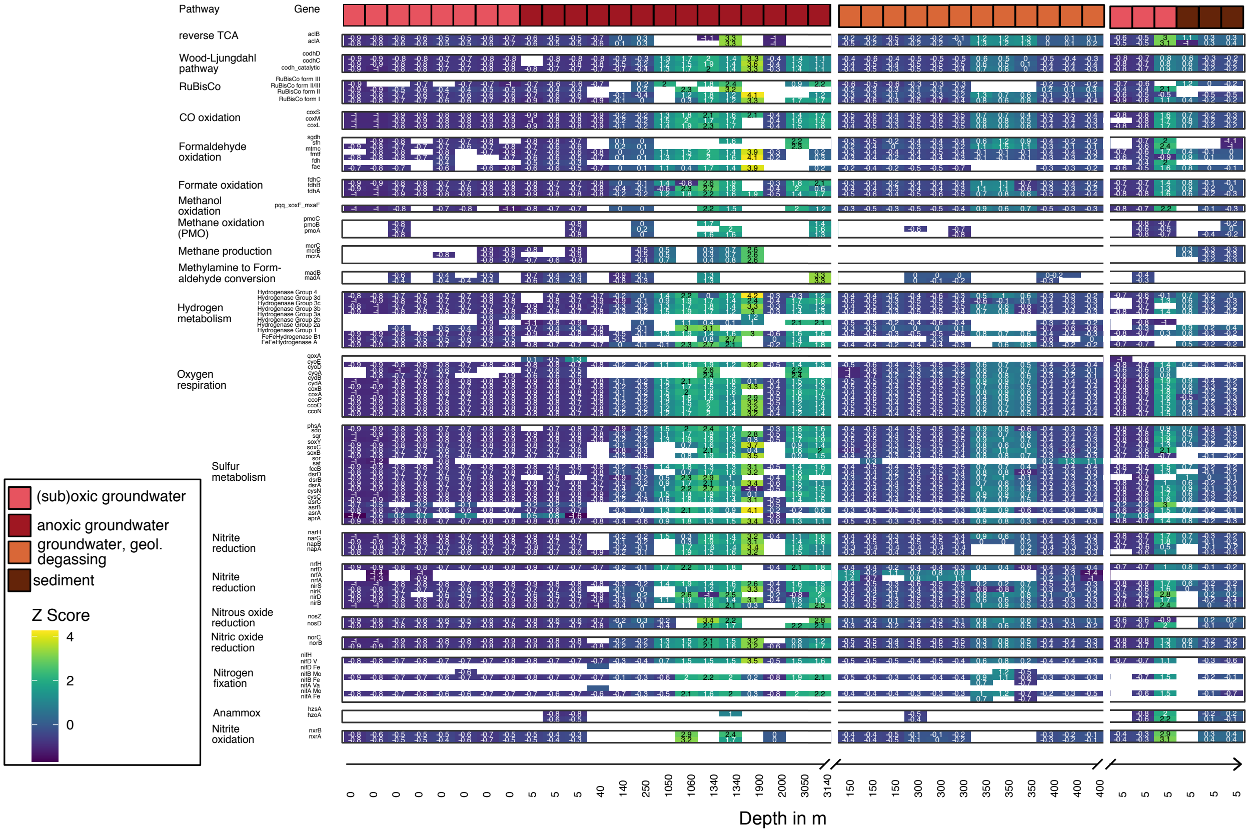

Supplementary Figure 7 | Correlation of ANI (A) and AAI (B) similarity of Alti-1 genomes with geographical distance. A: Correlation of average nucleotide identity (ANI) in dependency on the geographical distance between sampling sites. The elliptical geographical distance (as opposed to the Euclidian distance) was determined using the R package sp[33] and the ANI was determined using fastANI[34]. Pearson correlation was used to see whether the two variables show a dependency. **B:** Correlation of average amino acid identity (AAI) in dependency on the geographical distance between sampling sites. Red circles indicate the placement of individual Alti-1 comparisons in the ANI/AAI vs geographical distance space. The elliptical geographical distance (as opposed to the Euclidian distance) was determined using the R package sp and the AAI was determined using fastANI. Pearson correlation was used to see whether the two variables show a dependency.

A

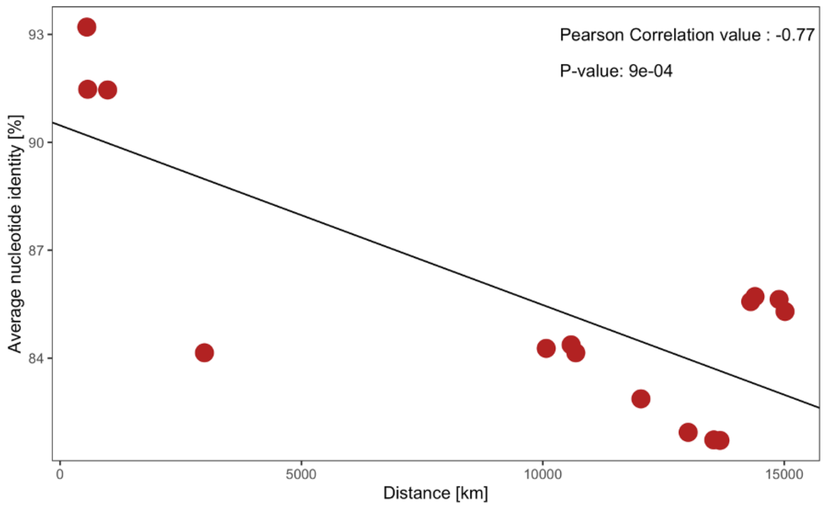

B

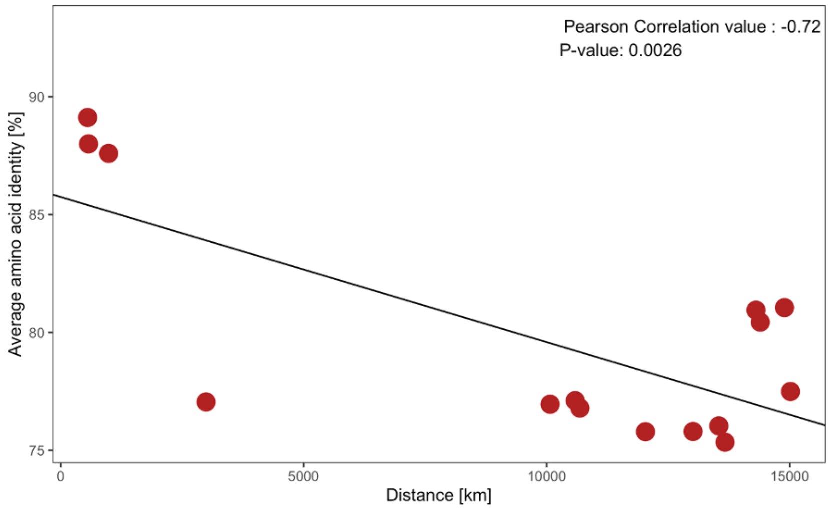

Supplementary Figure 8 | Phylogeny of Alti-1 genotypes based on 30 universal ribosomal proteins (5136 aa positions, IQTree JTTDCMut+F+G4) and using the Alti-2 genome IMC4 as the outgroup. Branch supports correspond to ultrafast bootstraps[35] (1000 replicates), the SH-aLRT[36] test (1000 replicates), and the approximate Bayes test[37] respectively.

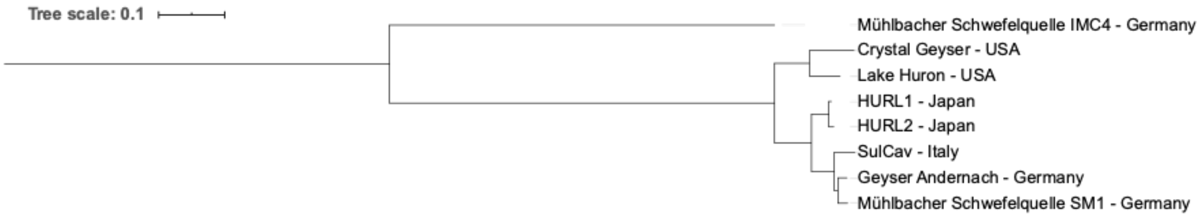

Supplementary Figure 9 | Comparison of minimal generation time values between *Ca.* Altiarcheales clades 1 and 2. Seven biologically independent Alti-1 species (boxplot statistics: median=7.82, upper quartile=7.86, lower quartile=7.38, upper whisker=7.88, lower whisker=6.80, minima= 6.80, maxima=10.525) and six biologically independent Alti-2 species (boxplot statistics: median=6.11, upper quartile=6.53, lower quartile=5.42, upper whisker=6.89, lower whisker=4.58, minima=4.58, maxima=6.89) were used to generate the boxplots. Box bounds represent 25 % and 75 % quartiles, respectively. Minimal generation times were averaged for species if multiple genomes, i.e., technical replicates, had been recovered for a specific population. The final used averages for this figure correspond to the average minimal generation times depicted in Figure 4.

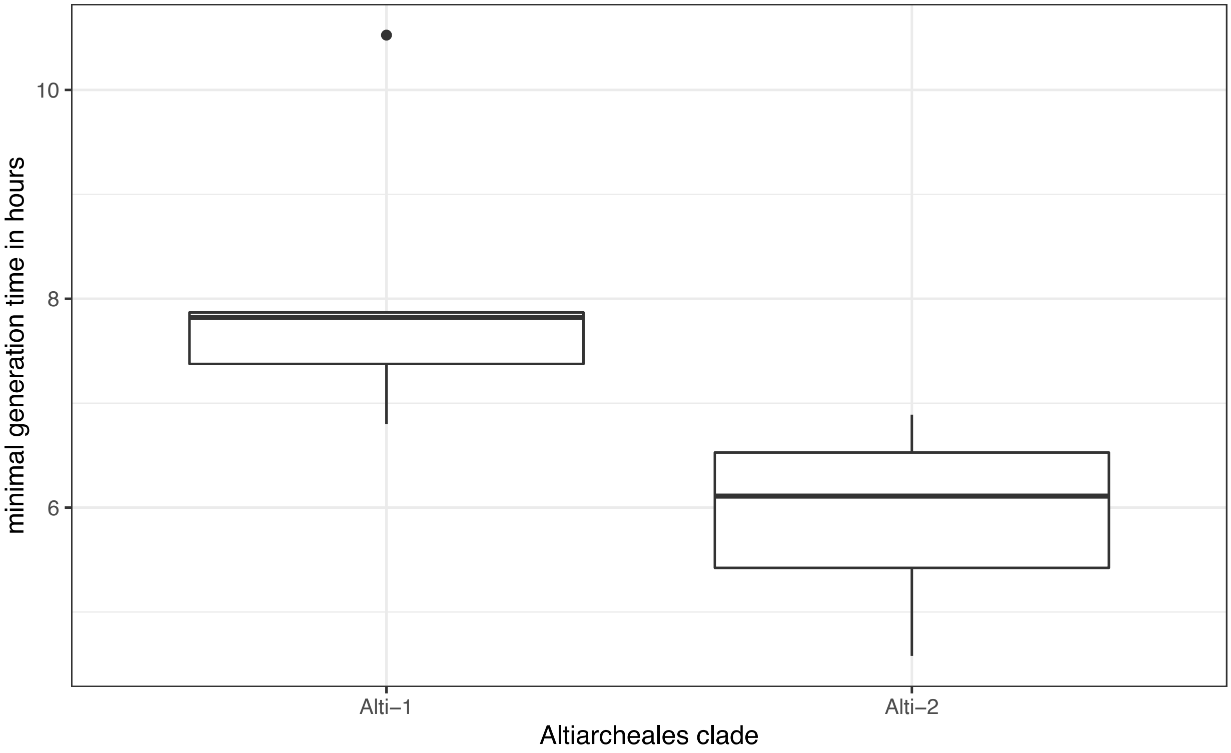

Supplementary Figure 10 | Correlation between Shannon-index and depth. Shannon index values were calculated using the R package vegan[38] from sequencing depth-normalized rpS3-scaffold abundances for each sample. The median for each sampling depth was computed and then correlated against the sampling site depth using Pearson correlation. The exact p-value is p = 0.01999.

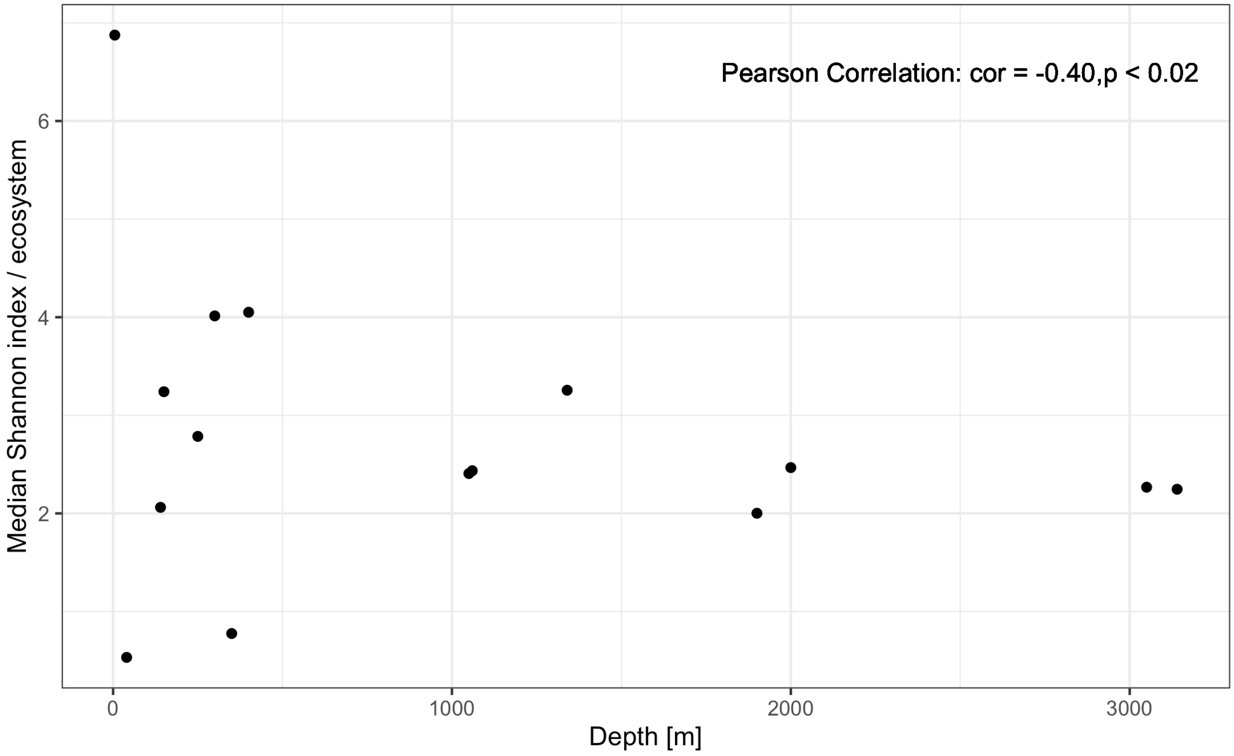

Supplementary Figure 11 | Protein similarity clustering networks of *Ca.* Altiarchaeales proteins with >= 80% similarity. A: Genomes are marked as large red circles with edges marked as black lines. Edge lengths are arbitrary. Genomes belonging to the Alti-1 subclade are enclosed by a square. The network was visualized in Cytoscape[39]. **B:** Presence (yellow) / absence (purple) heatmap of protein clusters (y-axis) across Altiarchaeales genomes (x-Axis) with an 80 % similarity cutoff and a hierarchical clustering dendrogram showing the overall relatedness of Altiarchaeles proteins.

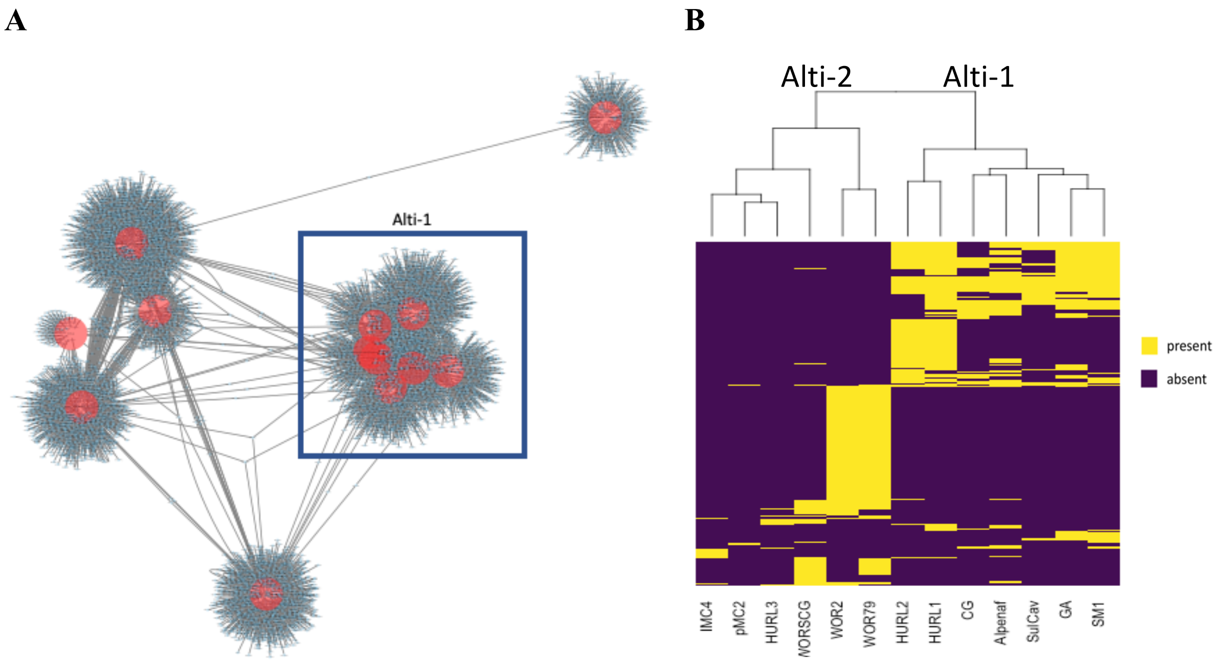

Supplementary Figure 12 | Abundance of recovered microorganisms in 2018 and 2019 based on read mapping on genomes of 2018. Coverages are shown as blue bars and coverages lower than one were removed as they resulted in negative values after log-scaling. The % alignment rate indicates the percentage of reads per sample that mapped to these genomes, i.e., how much of the ecosystem diversity is explained by these genomes.

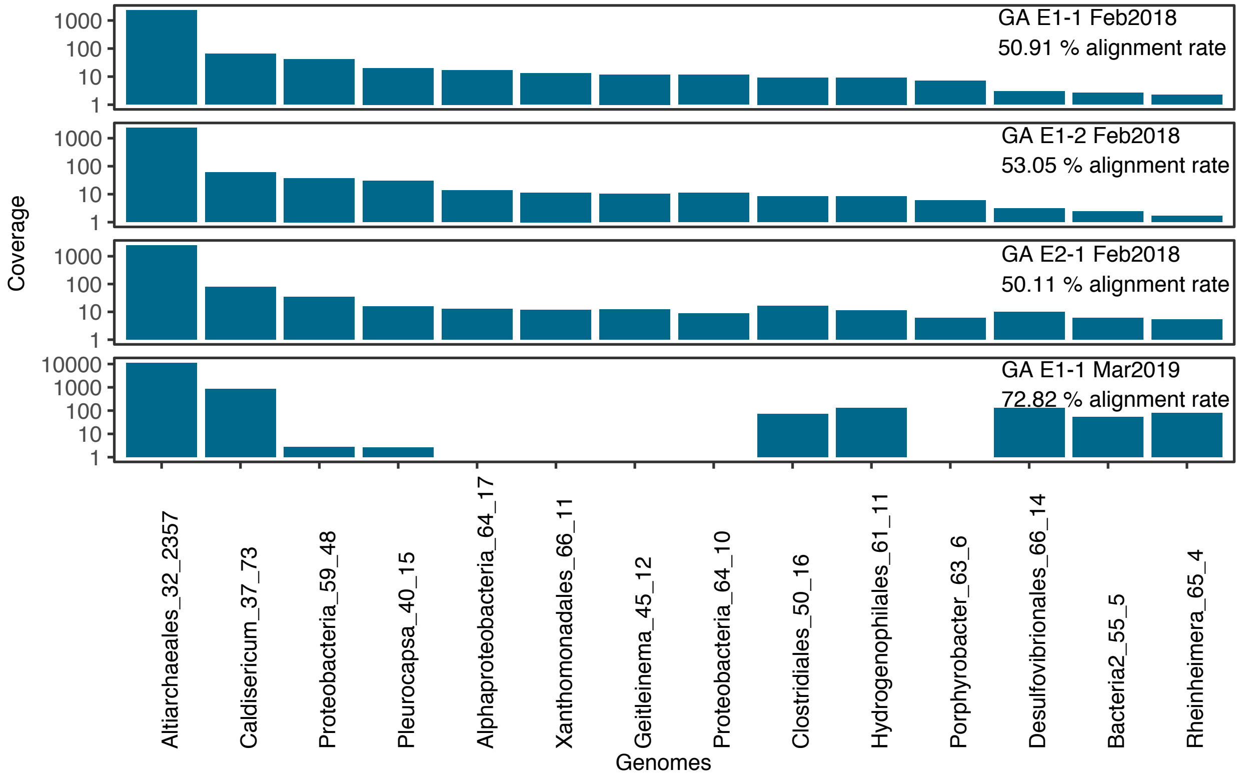

4. SUPPLEMENTARY TABLES

Supplementary Table 1 | Geochemical measurements of Geyser Andernach. Additional data can be found in Supplementary Data 4.

General characterization

Eruption interval	120 min
Eruption duration	6 (2004) – 30 (2018) min
Maximum eruption height	45 - 62 m
Eruption water volume	6 - 7m³ / eruption
Temperature water at surface	291 - 295 K
Water temperature in 240 m depth	299.65 K
CO_2 temperature at surface	293 – 295 K
Water Conductivity	12.800 - 13.600 µS
potentia hydrogenii (pH)	6.6 – 6.8
Chemical composition (averages)	
Sodium [Na^+]	2.750 mg l^{-1}
Kalium [K^+]	100 mg l^{-1}
Magnesium [Mg^{2+}]	340 mg l^{-1}
Calcium [Ca^{2+}]	300 mg l^{-1}
Manganese [Mn]	<1 mg l^{-1}
Iron [Fe]	10 mg l^{-1}
Chloride [Cl^-]	2.250 mg l^{-1}
Sulfate [SO_4^{2-}]	425 mg l^{-1}
Hydrogen carbonate [HCO_3^-]	5.700 mg l^{-1}
Carbon dioxide [CO_2]	1.500 mg l^{-1}

Gas composition[40]

Carbon dioxide [CO_2]	99.96 % (v/v)
Hydrogen [H_2]	2.4 ppmv
Oxygen [O_2]	0.008 % (v/v)
Nitrogen [N_2]	0.03 % (v/v)
Methane [CH_4]	BDL
Helium [He]	1.6 ppmv
Argon [Ar]	0.0007 % (v/v)

Chemical analysis of samples also investigated with metagenomics

Compound (unit)	AVG (STDEV)
Calcium Ca^{2+}(mM)	5.26 (0.21)
Magnesium Mg^{2+} (mM)	9.05 (0.27)
Potassium K^{+} (mM)	3.15 (0.86)
Ammonia NH_4^{+} (mM)	1.20 (0.35)
Sodium Na^{+} (mM)	73.05 (2.28)
Sulfate SO_4^{2-} (mM)	4.98 (0.39
Chloride Cl^{-} (mM)	70.58 (7.26)
Nitrate NO_3^{-} (mM)	BDL
Phosphate PO_4^{3-} (mM)	BDL
ferrous iron Fe^{2+} (mM)	BDL
Fe_{total} (µM)	486.11 (39.06)
H_2S (µM)	BDL
DOC (mg / l)	1.39 (0.31)

Supplementary Table 2 | Assembly statistics (all samples, all ecosystems).

Assembly	Publication of read dataset	# TB read bps	% reads mapped to assembly	# scaffold bps	# scaffolds >=1000 bps length	N50 scaffolds >=1000 bps length
GA_E1_1	This study	6.9	75	3.66E+08	1.08E+08	3090
GA_E1_2		6.7	76.9	3.33E+08	1.01E+08	3324
GA_E2_1		7.5	73	3.62E+08	1.04E+08	3927
Tomsk	Kadnikov et al. (2018)[41]	18.1	98.7	1.62E+08	1.38E+08	26124
AfrMine_BE2011	Lau et al. (2014)[42]	4	88.1	1.39E+08	8.53E+07	17953
AfrMine_BE2012		4.9	84.9	2.99E+08	1.81E+08	9193
AfrMine_DR5		5.2	92.6	1.24E+08	7.67E+07	6075
AfrMine_FI88		4.4	86.9	2.42E+08	1.45E+08	11589
AfrMine_MM5		2.7	95.6	4.10E+07	2.67E+07	5026
AfrMine_TT107		4.7	95.9	8.34E+07	5.86E+07	6592
AfrMine_TT109		4.6	94.7	9.48E+07	7.31E+07	21066
HURL_140m	Hernsdorf et al. (2017)[43]	14	97.4	1.79E+08	1.29E+08	13736
HURL_250m		13.7	96	2.92E+08	1.91E+08	15318
IMS	Probst et al. (2014)[13]	40.3	93.4	2.41E+08	1.13E+08	4545
SulCav_AS07-7	Hamilton et al. (2015)[44]	79.6	95.1	1.66E+09	4.11E+08	6162
SulCav_FS08-3		47.6	87.9	4.91E+09	4.66E+08	2219
SulCav_GS09-5		44.8	92.8	2.68E+09	7.70E+08	4281
SulCav_PC08-64		37.4	92	2.47E+09	4.69E+08	3088
SulCav_PC08-66		36.2	87.9	3.52E+09	6.79E+08	2820

SulCav_PC08-3		46.3	92.4	1.62E+09	3.87E+08	4522
SulCav_GS10-10		48.3	90.9	3.70E+09	1.02E+09	3059
SulCav_FS06-10		76.9	96.1	1.16E+09	3.18E+08	3467
Rifle_sed1	Anantharaman et al. (2016)	7.2	32.4	8.40E+08	1.94E+08	2242
Rifle_sed2		14.6	45	1.62E+09	4.83E+08	2305
Rifle_sed3		14.8	32.3	1.57E+09	3.52E+08	2078
Rifle_o2low1		41.1	82.2	3.97E+09	2.07E+09	4522
Rifle_o2low2		37	75.6	3.80E+09	1.68E+09	3594
Rifle_o2low3		4.8	44.7	6.44E+08	1.60E+08	2607
Rifle_o2high1		49.5	85.7	4.26E+09	2.08E+09	4688
Rifle_o2high2		39.3	75.9	4.80E+09	1.89E+09	3564
Rifle_o2high3		37.2	71.2	4.69E+09	1.66E+09	3427
CG03_2015	Probst et al. (2018)[8]	21.6	91.5	1.58E+09	6.24E+08	3987
CG04_2015		23.1	92	1.65E+09	6.70E+08	5131
CG05_2015		20.3	91.8	1.44E+09	5.82E+08	4321
CG11_2015		19.2	90.6	1.48E+09	5.83E+08	3844
CG12_2015		18.2	90.7	1.58E+09	5.83E+08	3948
CG13_2015		17	90.7	1.37E+09	5.04E+08	4070
CG21_2015		19.4	94.7	7.95E+08	3.24E+08	3743
CG22_2015		23.2	93.5	1.18E+09	5.45E+08	4952
CG23_2015		17	93.3	9.19E+08	3.89E+08	4222

Supplementary Table 3 | Origin and accession numbers of *Ca.* Altiarchaeota genomes. Genomes marked with * were used in the phylogenomic analysis of *Ca.* Altiarchaea using 30 marker genes (Figure 4C). For the analysis of the metabolic potential, all of the listed genomes were used.

Genome	Origin	Accession number
GA21<br>GA11<br>GA12	This study	SAMN18220854*<br>SAMN18220852<br>SAMN18220853
SulCav	This study	SAMN22209398*
LH	This study	SAMN18221259*
HURL1	Hernsdorf et al. (2017)<br>Rahlff et al. (2020)[45]	GCA_002841105.1*<br>SAMN18220775
HURL2	Hernsdorf et al. (2017)<br>Rahlff et al. (2020)	GCA_002841095.1*<br>SAMN18220774
IMS_SM1	Probst et al. (2014)<br>Rahlff et al. (2020)	CCXY00000000.1*<br>SAMN18220766
CG	Probst et al. (2018)	GCA_002789105.1*<br>GCA_002785505.1<br>GCA_002762655.1<br>GCA_002791795.1<br>GCA_002783845.1<br>GCA_002789105.1<br>CG_SAG_2014_w16S_Altiarchaeum_hamiconexum1_33_1<br>CG_SAG_2014_w16S_Altiarchaeum_hamiconexum2_33_1<br>CG_SAG_2014_w16S_Altiarchaeum_hamiconexum3_33_1<br>CG_SAG_2014_w16S_Altiarchaeum_hamiconexum4_33_1<br>CG_SAG_2014_w16S_Altiarchaeum_hamiconexum5_33_1<br>CG_SAG_2014_w16S_Altiarchaeum_hamiconexum6_33_1<br>CG_SAG_2014_w16S_Altiarchaeum_hamiconexum7_33_1
	Probst et al. (2017)[46]	CG1_02_FULL_Cand_Altiarchaeum_hamiconexum_SM1_33_652_curated<br>CG2_30_FULL_Ca_Altiarchaeum_hamiconexum_SM1_32_4952_curated<br>CG2_30_SUB100_Altiarchaeum_32_3053_curated<br>CG2_30_SUB10_Cand_Altiarchaeum_hamiconexum_32_499_curated
WOR2	Bird et al. (2016)	GCA_001723855.1
WORSCG	Bird et al. (2016)	GCA_001723845.1
WOR79	Bird et al. (2016)	GCA_001723835.1
pMC2	Rinke et al. (2013)	GCA_000402775.1
IMS_IMC4	Probst et al. (2014)	MCBF00000000.1*
HURL3	Hernsdorf et al. (2017)	GCA_002842715.1

Supplementary Table 4 | Data on subsurface samples and ecosystems analyzed in this study containing sampling sites, depth and references. For assembly stats of metagenomes please see Supplementary Table 2; for statistics of reconstructed genomes please see Supplementary Data 1.

**Ecosystem**	**Assembly**	**Publication of read dataset**	**Accession number [SRA, BioSample or BioProject]**	**Depth**
GA	GA_E1_1	This study	SAMN14680028	350 m
GA	GA_E1_2		SAMN14680029	
GA	GA_E2_1		SAMN14680030	
Tomsk	Tomsk	Kadnikov et al. (2018)	SRR7102746	2000 m
BE	AfrMine_BE2011	Lau et al. (2014)	SRR13164376	1339 m
BE	AfrMine_BE2012		SRR13164375	1339 m
DR5	AfrMine_DR5		SRR13164374	1046 m
FI88	AfrMine_FI88		SRR13164373	1056 m
MM5	AfrMine_MM5		SRR13124528	1900 m
TT107	AfrMine_TT107		SRR13124526	3048 m
TT109	AfrMine_TT109		SRR13164372	3136 m
lHURL	HURL_140m	Hernsdorf et al. (2017)	PRJNA321556	140 m
dHURL	HURL_250m			250 m
IMS	IMS	Probst et al. (2014	SRR1534154	40 m
AS	SulCav_AS07-7	Hamilton et al. (2015)	SRR1559028	0 m
FS	SulCav_FS08-3		SRR1560849	
GS	SulCav_GS09-5		SRR1560848	
PC	SulCav_PC08-64		SRR1560064	
PC	SulCav_PC08-66		SRR1559230	
PC	SulCav_PC08-3		SRR1560850	
GS	SulCav_GS10-10		SRR1559353	
FS	SulCav_FS06-10		SRR1560266	
sed	Rifle_sed1	Anantharaman et al. (2016)	SRX1085346	5 m
sed	Rifle_sed2		SRX1085347	
sed	Rifle_sed3		SRX1085348	
lO2	Rifle_o2low1		SRX1085356	
lO2	Rifle_o2low2		SRX1085358	
lO2	Rifle_o2low3		SRX1085360	
hO2	Rifle_o2high1		SRX1085354	
hO2	Rifle_o2high2		SRX1085350	
hO2	Rifle_o2high3		SRX1085352	
mCG	CG03_2015	Probst et al. (2018)	SRS2524942	250 m

mCG	CG04_2015		SRS2524959	
mCG	CG05_2015		SRS2524997	
dCG	CG11_2015		SRS2525260	400 m
dCG	CG12_2015		SRS2525262	
dCG	CG13_2015		SRS2525263	
sCG	CG21_2015		SRS2525667	150 m
sCG	CG22_2015		SRS2525670	
sCG	CG23_2015		SRS2525668	

Supplementary Table 5 | Median iRep values per sample and depth. Mean iRep for each genome per ecosystem values were used. Samples are ordered by depth. SulCav[44], IMS[13], AfrMine[47,42] and Tomsk[41] are publicly available datasets that were leveraged to bin new genomes and published genomes from Rifle[10], HURL[43] and CG[8] were used in this study after dereplication using dRep if not already performed on the available genomes.

Ecosystem	Sample	Depth	Median iRep
SulCav	AS	0	1.348
SulCav#	FS	0	1.395
SulCav*	PC	0	1.473
SulCav#	GS	0	1.407
Rifle*	hO2	5	1.332
IMS	IMS	40	1.336
HURL	sHURL	140	1.411
HURL	dHURL	250	1.344
AfrMine	DR5	1050	1.276
AfrMine	FI88	1060	1.290
AfrMine#	BE	1340	1.261
AfrMine	MM5	1900	1.225
Tomsk	Tomsk	2000	1.191
AfrMine	TT107	3050	1.205
AfrMine	TT109	3140	1.258
CG*	sCG	150	1.562
CG*	mCG	300	1.527
GA*	GA	350	1.396
CG*	dCG	400	1.540
Rifle*	lO2	5	1.347
Rifle*	Sed	5	1.588

*sample exists as triplicates, grouped for Fig. 2, separate for Fig. 3

#sample exists as duplicates, grouped for Fig. 2, separate for Fig. 3

Supplementary Table 6 | Pearson Correlations of iRep index values of genomes with specific metabolic capacities across depth. P-values were adjusted for multiple testing according to the Benjamini-Hochberg method.

High CO_2 ecosystems excluded (referred to in main text)

	Adjusted p-values	Correlation-values
Arsenate reduction/oxidation	5.68E-08	-0.42
C_1 compounds utilization	2.59E-10	-0.45
Carbon fixation	5.17E-11	-0.48
Hydrogen metabolism	3.99E-22	-0.45
Sulfur oxidation	2.85E-13	-0.46
Selenate reduction	2.15E-05	-0.48
Halogens breakdown / perchlorate reduction	2.82E-05	-0.39
Metals oxidation/reduction	0.145600971	-0.37
Nitrogen metabolism	3.62E-24	-0.42
Oxygen respiration	5.84E-26	-0.43
Sulfur reduction	0.019817469	-0.31
Carbon monoxide oxidation	4.64E-06	-0.43
Urea utilization	0.19515825	-0.27
Methane oxidation	0.141004585	-0.98

High CO_2 ecosystems included

	Adjusted p-values	Correlation-values
Arsenate reduction/oxidation	2.27E-08	-0.35
C_1 compounds utilization	3.89E-12	-0.41
Carbon fixation	9.25E-10	-0.39
Hydrogen metabolism	3.81E-29	-0.45
Sulfur oxidation	3.05E-13	-0.46
Selenate reduction	7.24E-06	-0.43
Halogens breakdown / perchlorate reduction	3.00E-06	-0.36
Metals oxidation/reduction	0.055037708	-0.33
Nitrogen metabolism	4.62E-26	-0.38
Oxygen respiration	9.04E-29	-0.39
Sulfur reduction	0.021233003	-0.31
Carbon monoxide oxidation	4.45E-07	-0.41
Urea utilization	0.052759627	-0.33
Methane oxidation	0.129494006	-0.98

Supplementary Table 7 | Two-group significance (high vs non-high CO_2 ecosystems) testing results of metabolic potentials in assemblies. The read-normalized abundances of metabolic pathways of high CO_2 ecosystems were compared using two-sided Welch's t-test and the two-sided Kruskal-Wallis test. P-values were adjusted using the Benjamini-Hochberg method.

**Metabolism**	**t-test 2-independent group P-values**	**Kruskal-Wallis group comparison**
Hydrogen metabolism	0.86	0.62
Oxygen respiration	0.28	0.23
Sulfur metabolism	0.86	0.97
rTCA	0.86	0.23
Carbon monoxide oxidation	0.86	0.11
Wood Lijungdahl pathway	0.86	0.38
Formaldehyde oxidation	0.27	0.23
Formate oxidation	0.86	0.85
Methylamine to formaldehyde	0.86	0.85
Methanol oxidation	0.97	0.39
Nitrate reduction	0.86	0.38
N_2 fixation	0.48	0.67
Nitric oxide reduction	0.86	0.67
Nitrite reduction	0.02	0.0006
Nitrous oxide reduction	0.28	0.62
Methane oxidation (PMO)	0.27	0.06
CBB pathway	0.27	0.85
Nitrite oxidation	0.44	0.05

Supplementary Table 8 | Hidden Markov models used for the prediction of the metabolic potential. The HMMs and supplied scores originate from Anantharaman et al. (2016)[10]. Hidden Markov-models with an associated score of 1 (HMMs for the propionyl-CoA-synthase/malonyl-CoA-reductase indicative of the 3-hydroxy-proprionate cycle as well as the 4-hydroxybutyryl-CoA synthetase and dehydratase indicative of the 3-hydroxyproprionate / 4-Hydroxybutyrate cycle) were removed as the low threshold did not allow for an accurate prediction of these enzymes. The HMM octR_TIGR04315 specific for the Octaheme cytochrome of type c from Shewanella was also excluded.

Hidden Markov-Model	Pathway	Score threshold
fccB_PF09242	Sulfide oxidation	266
sulfide_quinone _oxidoreductase_sqr	Sulfide oxidation	300
dsrD_PF08679	Sulfite reduction	50
dsrA_TIGR02064	Sulfur reduction	223
dsrB_TIGR02066	Sulfur reduction	205
sor_PF07682	Sulfur oxidation	300
sulfur_dioxygenase_sdo	Sulfur oxidation	120

soxB_TIGR04486	Thiosulfate oxidation	375
soxY_TIGR04488	Thiosulfate oxidation	125
soxC_TIGR04555	Thiosulfate oxidation	330
thiosulfate_reductase_phsA	Thiosulfate disproportionation	323
asrA_TIGR02910	Sulfate reduction	223
asrB_TIGR02911	Sulfate reduction	226
asrC_TIGR02912	Sulfate reduction	194
aprA_TIGR02061	Sulfate reduction	326
sat_TIGR00339	Sulfate reduction	181
cysC_TIGR00455	Sulfate reduction	133
cysN_TIGR02034	Sulfate reduction	327
FeFeHydrogenase_TIGR04105	Hydrogen metabolism	380
FeFeHydrogenase_TIGR02512	Hydrogen metabolism	300
Hydrogenase_Group_1	Hydrogen metabolism	411
Hydrogenase_Group_2a	Hydrogen metabolism	472
Hydrogenase_Group_2b	Hydrogen metabolism	555
Hydrogenase_Group_3a	Hydrogen metabolism	368
Hydrogenase_Group_3b	Hydrogen metabolism	303
Hydrogenase_Group_3c	Hydrogen metabolism	575
Hydrogenase_Group_3d	Hydrogen metabolism	541
Hydrogenase_Group_4	Hydrogen metabolism	430
pmoA_TIGR03080	Methane oxidation (PMO)	92
pmoB_TIGR03079	Methane oxidation (PMO)	202
pmoC_TIGR03078	Methane oxidation (PMO)	124
mcrA_TIGR03256	Methanogenesis	314
mcrB_TIGR03257	Methanogenesis	173
mcrC_TIGR03259	Methanogenesis	171
nifA_Fe_TIGR0186	Nitrogen fixation	873
nifB_Fe_TIGR02931	Nitrogen fixation	853
nifD_Fe_TIGR02929	Nitrogen fixation	124
nifA_Mo_TIGR01282	Nitrogen fixation	503
nifB_Mo_TIGR01286	Nitrogen fixation	414
nifA_Va_TIGR01860	Nitrogen fixation	822
nifB_V_TIGR02932	Nitrogen fixation	821
nifD_V_TIGR02930	Nitrogen fixation	122
nifH_TIGR01287	Nitrogen fixation	261
nitrite_oxidoreductase_nxrA	Nitrite oxidation	370

nitrite_oxidoreductase_nxrB	Nitrite oxidation	252
napA_TIGR01706	Nitrate reduction	472
NapB_PF03892	Nitrate reduction	23.9
narG_TIGR01580	Nitrate reduction	600
narH_TIGR01660	Nitrate reduction	348
nrfH_TIGR03153	Nitrite reduction	75
nrfA_PF02335	Nitrite reduction	57
nrfA_TIGR03152	Nitrite reduction	576
nrfD_TIGR03148	Nitrite reduction	300
nirB_TIGR02374	Nitrite reduction	442
nirD_TIGR02378	Nitrite reduction	69
nirK_TIGR02376	Nitrite reduction	169
nitrite_reductase_nirS	Nitrite reduction	200
nitric_oxide_reductase_norB	Nitric oxide reduction	79
nitric_oxide_reductase_norC	Nitric oxide reduction	50
nosD_TIGR04247	Nitrous oxide reduction	290
nosZ_TIGR04246	Nitrous oxide reduction	550
hydrazine_oxidase_hzoA	Anammox	325
hydrazine_synthase_hzsA	Anammox	466
coxA_TIGR02891 (Cytochrome *c caa*3)	Oxygen respiration	617
coxB_TIGR02866 (Cytochrome *c caa*3)	Oxygen respiration	144
ccoN_TIGR00780 (Cytochrome *c cbb*3)	Oxygen respiration	168
ccoO_TIGR00781 (Cytochrome *c cbb*3)	Oxygen respiration	62
ccoP_TIGR00782 (Cytochrome *c cbb*3)	Oxygen respiration	54
cyoA_TIGR01433 (Cytochrome *c bo*)	Oxygen respiration	218
cyoD_TIGR02847 (Cytochrome *c bo*)	Oxygen respiration	64
cyoE_TIGR01473 (Cytochrome *c bo*)	Oxygen respiration	138
cydA_PF01654 (Cytochrome *c bd*)	Oxygen respiration	118
cydB_TIGR00203 (Cytochrome *c bd*)	Oxygen respiration	88
qoxA_TIGR01432 (Cytochrome *c aa3*)	Oxygen respiration	204
qoxB_TIGR02882 (Cytochrome *c aa3*)	Oxygen respiration	954
methanol_dehydrogenase _pqq_xoxF_mxaF	Methanol oxidation	166
ndma_TIGR04266	Methanol oxidation	400
madA_TIGR02659	Methylamine to formaldehyde conversion	85
madB_TIGR02658	Methylamine to formaldehyde conversion	90
fdh_thiol_id_TIGR02819	Formaldehyde oxidation	425

sfh_TIGR02821	Formaldehyde oxidation	131
sgdh_TIGR02818	Formaldehyde oxidation	509
smdh_TIGR03451	Formaldehyde oxidation	512
fae_TIGR03126	Formaldehyde oxidation	31
fmtf_TIGR03119	Formaldehyde oxidation	94
mtmc_TIGR03120	Formaldehyde oxidation	147
fdhA_TIGR01591	Formate oxidation	610
fdhB_TIGR01582	Formate oxidation	152
fdhC_TIGR01583	Formate oxidation	56
carbon_monoxide_dehydrogenase_coxM	Carbon monoxide (CO) oxidation	198
carbon_monoxide_dehydrogenase_coxS	Carbon monoxide (CO) oxidation	184
carbon_monoxide_dehydrogenase _coxL_TIGR02416	Carbon monoxide oxidation	300
rubisco_form_I	CBB cycle	500
rubisco_form_II	CBB cycle	500
rubisco_form_II_III	CBB cycle	500
rubisco_form_III	CBB cycle	450
codhD_TIGR00381	Wood Ljungdahl pathway	197
codhC_TIGR00316	Wood Ljungdahl pathway	355
codh_catalytic_TIGR01702	Wood Ljungdahl pathway	210
acetate_citrate_lyase_aclA	Reverse TCA	215
acetate_citrate_lyase_aclB	Reverse TCA	177
ureC_TIGR01792	Urea breakdown	212
ureB_TIGR00192	Urea breakdown	40
ureA_TIGR00193	Urea breakdown	31
hdh_TIGR01428	Halogen breakdown	72
rdh_TIGR02486	Halogen breakdown	132
pcrA_TIGR03479	Perchlorate Reduction	559
pcrB_TIGR03478	Perchlorate Reduction	319
cld_PF06778	Chlorite Reduction	65
ars_ox_TIGR02694	Arsenite Oxidation	61
ars_ox_TIGR02693	Arsenite Oxidation	357
ars_thioredoxin_TIGR02691	Arsenite Reduction	112
ars_glutaredoxin_TIGR02689	Arsenite Reduction	113
asrC_TIGR00014	Arsenite Reduction	61

ygfM_TIGR03312	Selenate Reduction	87
sel_mo_TIGR03313	Selenate Reduction	526
ygfK_TIGR03315	Selenate Reduction	354
nthA_TIGR01323	Nitrile hydratase	239
nthB_TIGR03888	Nitrile hydratase	136
mtrA_TIGR03507	Metal Oxidation/Reduction	60
mtrC_TIGR03509	Metal Oxidation/Reduction	165

5. REFERENCES

1. Kallmeyer, J., Pockalny, R., Adhikari, R. R., Smith, D. C. & D'Hondt, S. Global distribution of microbial abundance and biomass in subseafloor sediment. *Proc. Natl. Acad. Sci. U. S. A.* **109**, 16213–16216 (2012).
2. Hug, L. A. *et al.* A new view of the tree of life. *Nat. Microbiol.* **1**, 16048 (2016).
3. Brown, C. T., Olm, M. R., Thomas, B. C. & Banfield, J. F. Measurement of bacterial replication rates in microbial communities. *Nat. Biotechnol.* **34**, 1256–1263 (2016).
4. Dick, G. J. *et al.* Community-wide analysis of microbial genome sequence signatures. *Genome Biol.* **10**, R85 (2009).
5. Wu, Y.-W., Simmons, B. A. & Singer, S. W. MaxBin 2.0: an automated binning algorithm to recover genomes from multiple metagenomic datasets. *Bioinformatics* **32**, 605–607 (2016).
6. Alneberg, J. *et al.* Binning metagenomic contigs by coverage and composition. *Nat. Methods* **11**, 1144–1146 (2014).
7. Sieber, C. M. K. *et al.* Recovery of genomes from metagenomes via a dereplication, aggregation and scoring strategy. *Nat. Microbiol.* **3**, 836–843 (2018).

8. Probst, A. J. *et al.* Differential depth distribution of microbial function and putative symbionts through sediment-hosted aquifers in the deep terrestrial subsurface. *Nat. Microbiol.* **3**, 328–336 (2018).
9. Olm, M. R., Brown, C. T., Brooks, B. & Banfield, J. F. dRep: a tool for fast and accurate genomic comparisons that enables improved genome recovery from metagenomes through de-replication. *ISME J.* **11**, 2864 (2017).
10. Anantharaman, K. *et al.* Thousands of microbial genomes shed light on interconnected biogeochemical processes in an aquifer system. *Nat. Commun.* **7**, 1–11 (2016).
11. Cline, J. D. Spectrophotometric Determination of Hydrogen Sulfide in Natural Waters1. *Limnol. Oceanogr.* **14**, 454–458 (1969).
12. Moissl, C., Rachel, R., Briegel, A., Engelhardt, H. & Huber, R. The unique structure of archaeal 'hami', highly complex cell appendages with nano-grappling hooks: Unique structure of archaeal 'hami'. *Mol. Microbiol.* **56**, 361–370 (2005).
13. Probst, A. J. *et al.* Biology of a widespread uncultivated archaeon that contributes to carbon fixation in the subsurface. *Nat. Commun.* **5**, 5497 (2014).
14. Bird, J. T., Baker, B. J., Probst, A. J., Podar, M. & Lloyd, K. G. Culture Independent Genomic Comparisons Reveal Environmental Adaptations for Altiarchaeales. *Front. Microbiol.* **7**, (2016).
15. Papke, R. T., Ramsing, N. B., Bateson, M. M. & Ward, D. M. Geographical isolation in hot spring cyanobacteria. *Environ. Microbiol.* **5**, 650–659 (2003).
16. Whitaker, R. J., Grogan, D. W. & Taylor, J. W. Geographic barriers isolate endemic populations of hyperthermophilic archaea. *Science* **301**, 976–978 (2003).

17. Liu, L. *et al.* High correlation between genotypes and phenotypes of environmental bacteria Comamonas testosteroni strains. *BMC Genomics* **16**, (2015).

18. Wright, S. Isolation by Distance. *Genetics* **28**, 114–138 (1943).

19. Wright, S. Isolation by Distance Under Diverse Systems of Mating. *Genetics* **31**, 39–59 (1946).

20. Malecot, G. Mathematics of heredity. *Math. Hered.* (1948).

21. Kimura, M. & Weiss, G. H. The Stepping Stone Model of Population Structure and the Decrease of Genetic Correlation with Distance. *Genetics* **49**, 561–576 (1964).

22. Maruyama, M. On automorphism groups of ruled surfaces. *J. Math. Kyoto Univ.* **11**, 89–112 (1971).

23. Martiny, J. B. H. *et al.* Microbial biogeography: putting microorganisms on the map. *Nat. Rev. Microbiol.* **4**, 102–112 (2006).

24. Hanson, C. A., Fuhrman, J. A., Horner-Devine, M. C. & Martiny, J. B. H. Beyond biogeographic patterns: processes shaping the microbial landscape. *Nat. Rev. Microbiol.* **10**, 497–506 (2012).

25. Gaisin, V. A. *et al.* Biogeography of thermophilic phototrophic bacteria belonging to Roseiflexus genus. *FEMS Microbiol. Ecol.* **92**, (2016).

26. Varliero, G., Bienhold, C., Schmid, F., Boetius, A. & Molari, M. Microbial Diversity and Connectivity in Deep-Sea Sediments of the South Atlantic Polar Front. *Front. Microbiol.* **10**, (2019).

27. Lovley, D. R. & Chapelle, F. H. Deep subsurface microbial processes. *Rev. Geophys.* **33**, 365–381 (1995).

28. Schreiber, U. *et al.* Organic compounds in fluid inclusions of Archean quartz—Analogues of prebiotic chemistry on early Earth. *PLOS ONE* **12**, e0177570 (2017).

29. Rodriguez-R, L. M., Gunturu, S., Tiedje, J. M., Cole, J. R. & Konstantinidis, K. T. Nonpareil 3: Fast Estimation of Metagenomic Coverage and Sequence Diversity. *mSystems* **3**, (2018).

30. Magnabosco, C. *et al.* The biomass and biodiversity of the continental subsurface. *Nat. Geosci.* **11**, 707–717 (2018).

31. Vieira-Silva, S. & Rocha, E. P. C. The Systemic Imprint of Growth and Its Uses in Ecological (Meta)Genomics. *PLOS Genet.* **6**, e1000808 (2010).

32. Weissman, J. L., Hou, S. & Fuhrman, J. A. Estimating maximal microbial growth rates from cultures, metagenomes, and single cells via codon usage patterns. *bioRxiv* 2020.07.25.221176 (2020) doi:10.1101/2020.07.25.221176.

33. Pebesma, E. & Bivand, R. Classes and Methods for Spatial Data in R. *R News* **5**, (2005).

34. Jain, C., Rodriguez-R, L. M., Phillippy, A. M., Konstantinidis, K. T. & Aluru, S. High throughput ANI analysis of 90K prokaryotic genomes reveals clear species boundaries. *Nat. Commun.* **9**, (2018).

35. Hoang, D. T., Chernomor, O., von Haeseler, A., Minh, B. Q. & Vinh, L. S. UFBoot2: Improving the Ultrafast Bootstrap Approximation. *Mol. Biol. Evol.* **35**, 518–522 (2018).

36. Guindon, S. *et al.* New algorithms and methods to estimate maximum-likelihood phylogenies: assessing the performance of PhyML 3.0. *Syst. Biol.* **59**, 307–321 (2010).

37. Anisimova, M., Gil, M., Dufayard, J.-F., Dessimoz, C. & Gascuel, O. Survey of Branch Support Methods Demonstrates Accuracy, Power, and Robustness of Fast Likelihood-based Approximation Schemes. *Syst. Biol.* **60**, 685–699 (2011).

38. Oksanen, J. *et al.* vegan: Community Ecology Package. (2012).

39. Shannon, P. *et al.* Cytoscape: a software environment for integrated models of biomolecular interaction networks. *Genome Res.* **13**, 2498–2504 (2003).

40. Bräuer, K., Kämpf, H., Niedermann, S. & Strauch, G. Indications for the existence of different magmatic reservoirs beneath the Eifel area (Germany): A multi-isotope (C, N, He, Ne, Ar) approach. *Chem. Geol.* **356**, 193–208 (2013).

41. Kadnikov, V. V. *et al.* A metagenomic window into the 2-km-deep terrestrial subsurface aquifer revealed multiple pathways of organic matter decomposition. *FEMS Microbiol. Ecol.* **94**, (2018).

42. Lau, M. C. Y. *et al.* Phylogeny and phylogeography of functional genes shared among seven terrestrial subsurface metagenomes reveal N-cycling and microbial evolutionary relationships. *Front. Microbiol.* **5**, (2014).

43. Hernsdorf, A. W. *et al.* Potential for microbial H 2 and metal transformations associated with novel bacteria and archaea in deep terrestrial subsurface sediments. *ISME J.* **11**, 1915–1929 (2017).

44. Hamilton, T. L., Jones, D. S., Schaperdoth, I. & Macalady, J. L. Metagenomic insights into S(0) precipitation in a terrestrial subsurface lithoautotrophic ecosystem. *Front. Microbiol.* **5**, (2015).

45. Rahlff, J. *et al.* Lytic archaeal viruses infect abundant primary producers in Earth's crust. *Nat. Commun.* **12**, 4642 (2021).

46. Probst, A. J. *et al.* Genomic resolution of a cold subsurface aquifer community provides metabolic insights for novel microbes adapted to high CO2 concentrations. *Environ. Microbiol.* **19**, 459–474 (2017).

47. Magnabosco, C. *et al.* A metagenomic window into carbon metabolism at 3 km depth in Precambrian continental crust. *ISME J.* **10**, 730 (2016).
