# Supplementary material for: Genetic diversity in terrestrial subsurface ecosystems impacted by geological degassing
Source: Nat Commun. 2022 Jan 12;13:284. doi: 10.1038/s41467-021-27783-7 (PMC8755723; doi:10.1038/s41467-021-27783-7)
Supplement: Supplementary file 3 — Description of Additional Supplementary Files [file 41467_2021_27783_MOESM3_ESM.pdf]

Description of Additional Supplementary Files

File Name: Supplementary Data 1
Description: Recovered genomes, consensus taxonomy, predicted minimal generation times, completeness and contamination. Genome statistics for dereplicated genomes from the Crystal Geyser along with their completeness and contamination are depicted in Probst et al. (2018).

File Name: Supplementary Data 2
Description: Mean and maximum replication index values of bacteria across subsurface ecosystems. Genomes of samples with biological replicates were dereplicated using dRep and their replication index values were dereplicated by calculating both the mean iRep value across the replicates as well as the maximum iRep value across the replicates.

File Name: Supplementary Data 3
Description: Genes used to conduct the Ca. Altiarchaea genome comparison (Fig. 5). A previously used set of annotated genes with the therein used numbering scheme (Supplementary Table 2 in Probst et al. (2014)) was used as the basis for the comparison and expanded using gene annotations from METABOLIC.

File Name: Supplementary Data 4
Description: Additional geochemical measurements of various laboratories from 1904 to 2004. Measurements were done according to the german TrinwV-GW (drinking water guidelines).

File Name: Supplementary Data 5
Description: Tree file of all bacterial genomes using 16 ribosomal proteins:

File Name: Supplementary Data 6

Description: Altiarchaeles 16S rRNA gene-based phylogeny. One representative of each DPANN phylum was used as the outgroup.

File Name: Supplementary Data 7-19
Description: Phylogenetic trees of Ca. Altiarchaea genes for detection of Horizontal Gene Transfer. Branches of the trees corresponding to Altiarchaeota Alti-1 and Alti-2 clades were colored using Dendroscope with red and blue, respectively.
